# Supplementary material for: Mutational escape from the polyclonal antibody response to SARS-CoV-2 infection is largely shaped by a single class of antibodies
Source: bioRxiv. 2021 Mar 18:2021.03.17.435863. Preprint. [Version 1] doi: 10.1101/2021.03.17.435863 (PMC7987015; doi:10.1101/2021.03.17.435863)
Supplement: 1 [file NIHPP2021.03.17.435863-supplement-1.pdf]

bioRxiv preprint doi: https://doi.org/10.1101/2021.03.17.435863; this version posted March 18, 2021. The copyright holder for this preprint (which was not certified by peer review) is the author/funder, who has granted bioRxiv a license to display the preprint in perpetuity. It is made available under aCC-BY 4.0 International license.

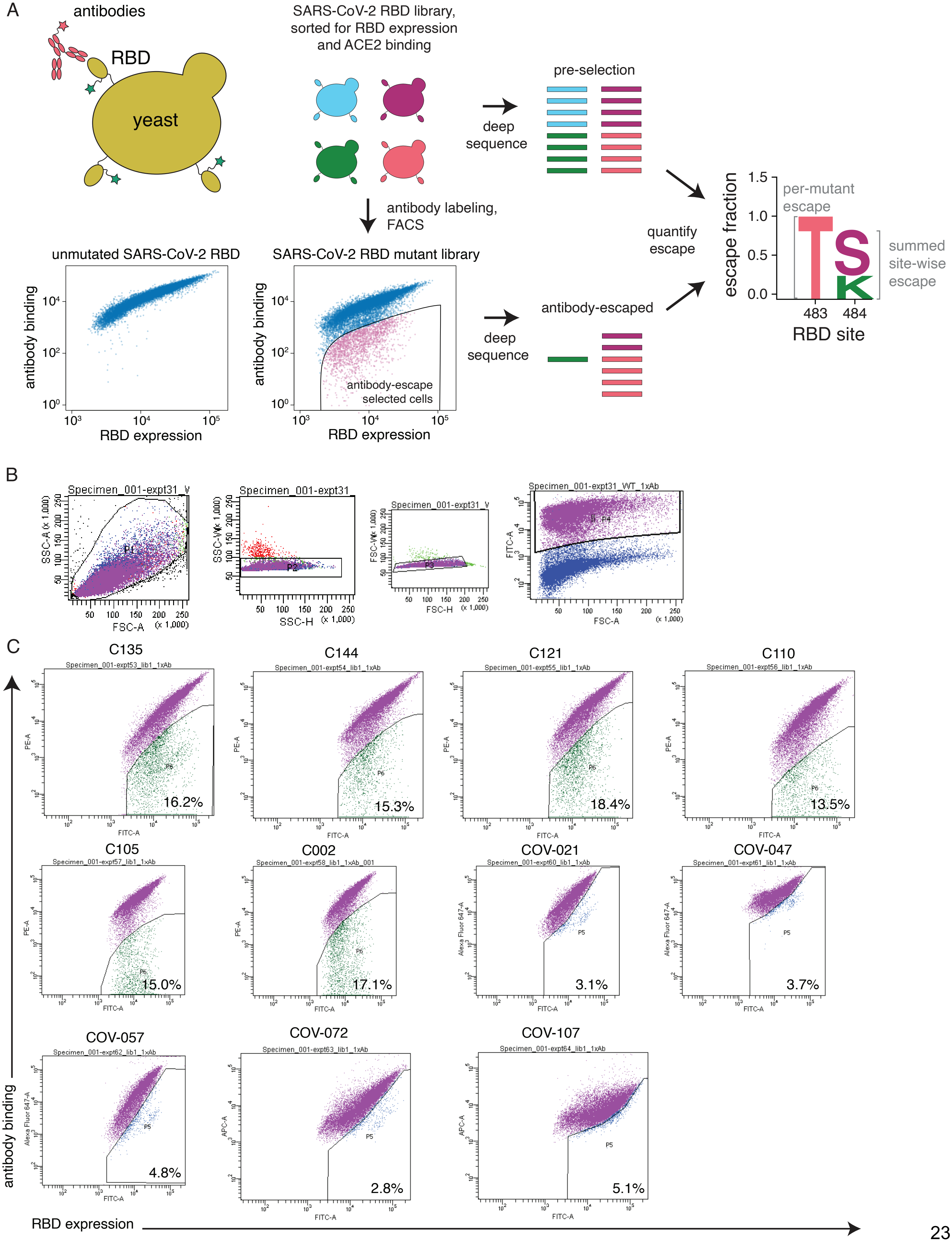

bioRxiv preprint doi: https://doi.org/10.1101/2021.03.17.435863; this version posted March 18, 2021. The copyright holder for this preprint (which was not certified by peer review) is the author/funder, who has granted bioRxiv a license to display the preprint in perpetuity. It is made available under aCC-BY 4.0 International license.

Figure S1. **Approach for mapping RBD mutations that reduce binding by monoclonal antibodies or polyclonal plasma, related to Figure 1. (A)** The RBD is expressed on the surface of yeast. Flow cytometry is used to quantify both RBD expression (via a C-terminal MYC tag) and antibody binding to the RBD protein expressed on the surface of each yeast cell. A library of yeast expressing different RBD mutants were incubated with antibodies or plasma and binding was detected using a IgG or IgA+IgG+IgM secondary antibody for monoclonal antibodies or polyclonal plasma, respectively. We then used FACS to enrich for cells expressing RBD that bound reduced levels of antibody, and deep sequencing to quantify the frequency of each mutation in the initial and "antibody escape" cell populations. We quantified the effect of each mutation as the "escape fraction," which represents the fraction of cells expressing RBD with that mutation that fell in the "antibody escape" FACS bin. Escape fractions are represented in logo plots, with the height of each letter proportional to the effect of that amino-acid mutation on antibody binding. The site-level escape metric is the sum of the escape fractions of all mutations at a site. Note that both experimental and computational filtering steps were used to remove RBD mutants that were misfolded or completely unable to bind the ACE2 receptor (see **Methods**). **(B)** Representative plots of nested FACS gating strategy used for all plasma selection experiments to select for single cells (SSC-A vs. FSC-A, SSC-W vs. SSC-H, and FSC-W vs. FSC-H) that also express RBD (FITC-A vs. FSC-A). **(C)** FACS gating strategy for one of two independent libraries to select cells expressing RBD mutants with reduced binding by monoclonal antibodies or polyclonal plasma (cells in blue). Gates were set manually during sorting. Different strategies were used for monoclonal antibodies vs. polyclonal plasma. For monoclonal antibodies, selection gates were set to capture up to 95% of yeast cells expressing unmutated RBD, stained with an antibody concentration 100x lower than that used for library staining. For polyclonal plasma, selection gates were set to capture 3-6% of the RBD+ library. The same gate was set for both independent libraries stained with each plasma, and the FACS scatter plots looked qualitatively similar between the two libraries. For information on the fraction of library cells that fall into each selection gate, see **Supplementary Table 2**.

bioRxiv preprint doi: https://doi.org/10.1101/2021.03.17.435863; this version posted March 18, 2021. The copyright holder for this preprint (which was not certified by peer review) is the author/funder, who has granted bioRxiv a license to display the preprint in perpetuity. It is made available under aCC-BY 4.0 International license.

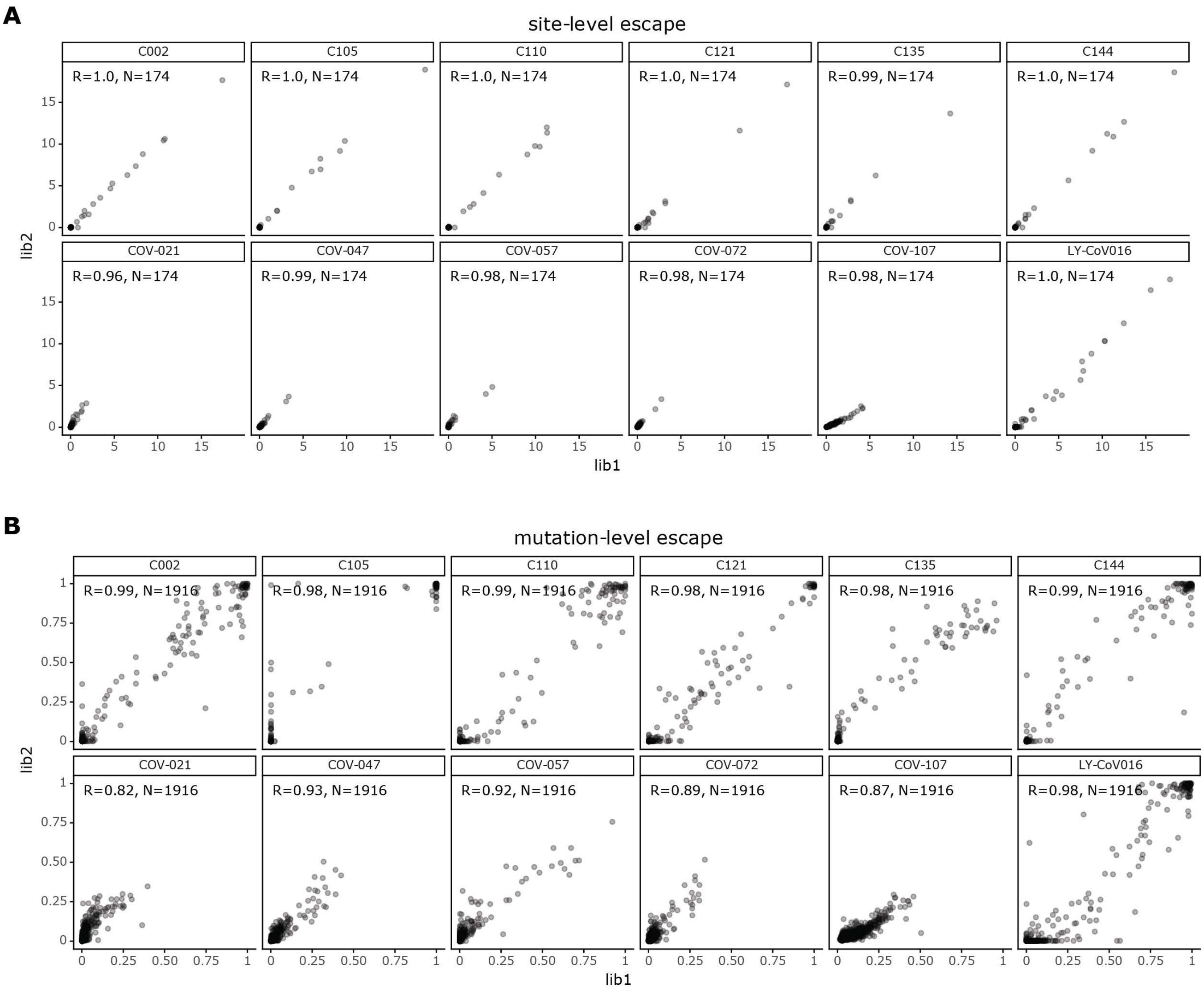

Figure S2. Correlations between replicates for site- and mutation-level escape metrics, related to Figure 1. (A) Correlation plots of site-level escape for each of the two independent RBD mutant libraries for each antibody or plasma. Each point represents one site in the RBD. **(B)** Correlation plots of mutation-level escape for each of the two independent RBD mutant libraries for each antibody or plasma. In this plot, each point represents a different mutation.

bioRxiv preprint doi: https://doi.org/10.1101/2021.03.17.435863; this version posted March 18, 2021. The copyright holder for this preprint (which was not certified by peer review) is the author/funder, who has granted bioRxiv a license to display the preprint in perpetuity. It is made available under aCC-BY 4.0 International license.

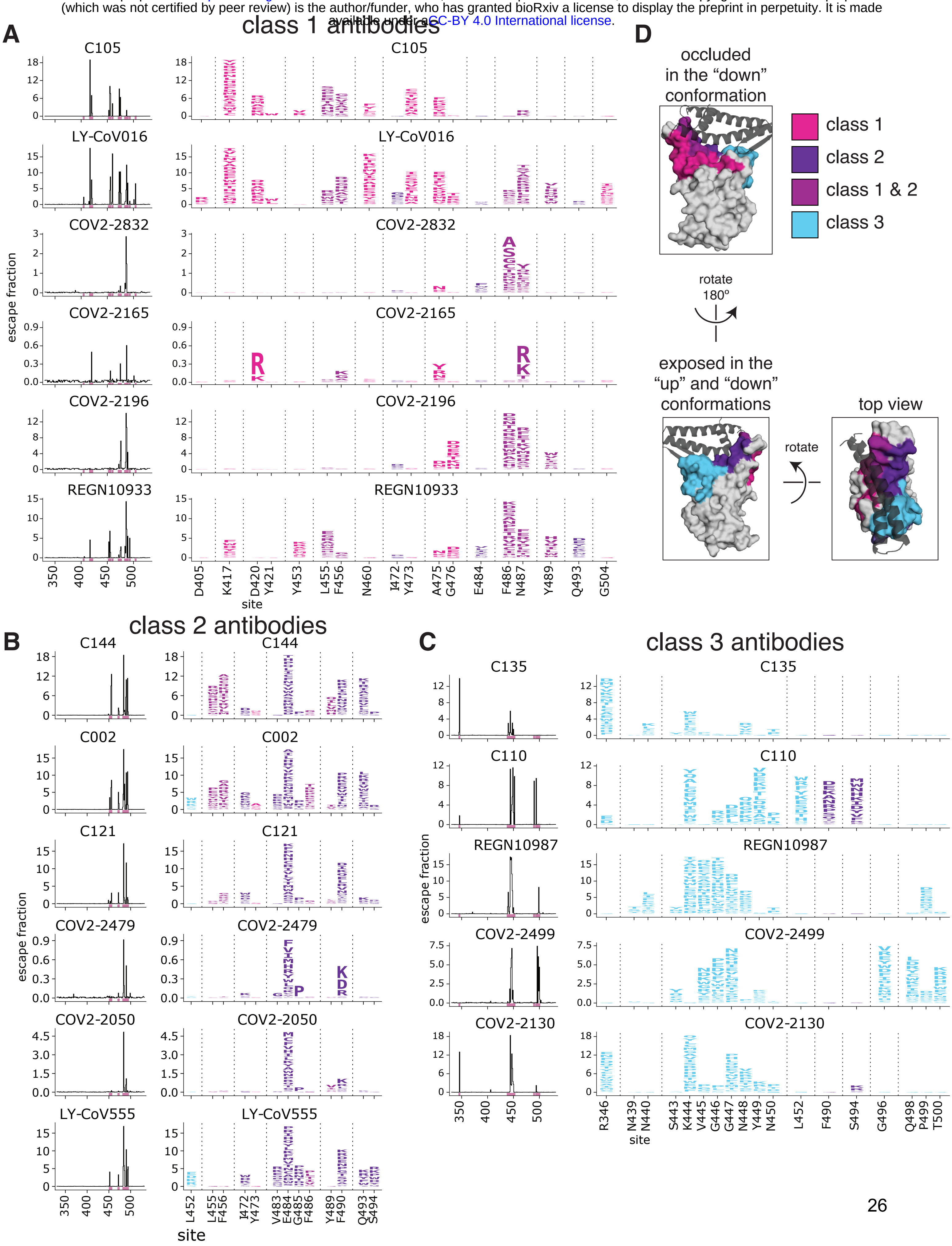

bioRxiv preprint doi: https://doi.org/10.1101/2021.03.17.435863; this version posted March 18, 2021. The copyright holder for this preprint (which was not certified by peer review) is the author/funder, who has granted bioRxiv a license to display the preprint in perpetuity. It is made available under aCC-BY 4.0 International license.

Figure S3. Escape maps for class 1, 2, or 3 antibodies we have profiled here or in previous studies, related to Figure 1. Escape maps for **(A)** class 1, **(B)** class 2, or **(C)** class 3 antibodies shown in **Figure 1C**. All escape maps were previously generated[30,33,35,36] except for C105, C144, C002, C121, C135, and C110 which are new to this study. Different sets of key sites are shown for each of the three antibody classes (see **Methods**). Sites are colored by RBD epitope as in **Figure 1,** also shown in panel **(D)**. For the escape maps of class 4 antibodies shown in **Figure 1C**, see Greaney et al. (2021)[30].

bioRxiv preprint doi: https://doi.org/10.1101/2021.03.17.435863; this version posted March 18, 2021. The copyright holder for this preprint (which was not certified by peer review) is the author/funder, who has granted bioRxiv a license to display the preprint in perpetuity. It is made available under aCC-BY 4.0 International license.

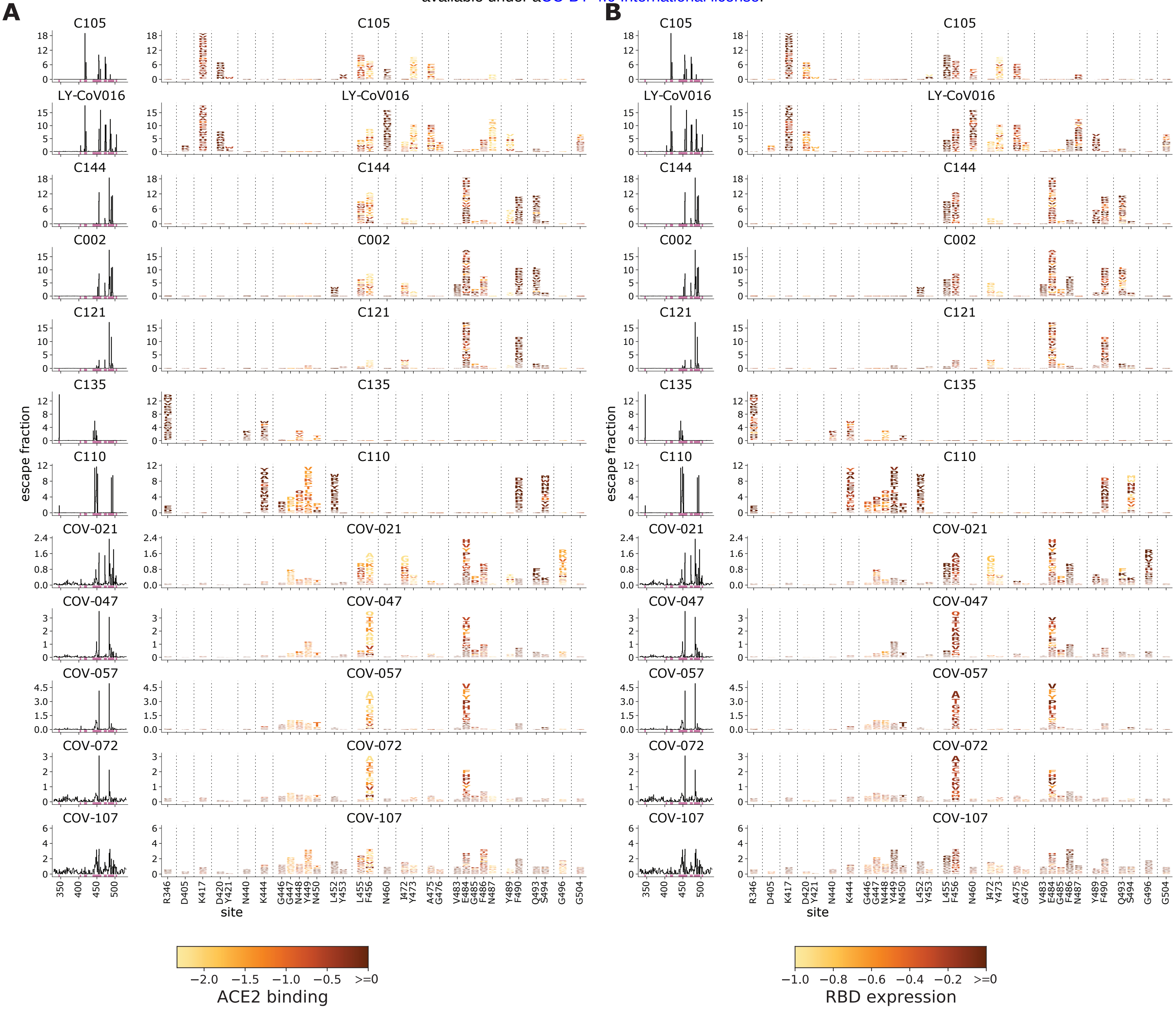

Figure S4. Logo plots colored according to effects of mutations on ACE2 binding and RBD expression, **related to Figures 1, 3, and 4**. Logo plots show the escape fractions of each mutation at key sites (any site called as a site of "strong escape" for any antibody or plasma). Letters are colored by how mutations affect RBD affinity for ACE2 or RBD expression as measured via yeast display[32], with yellow indicating poor affinity or expression and brown indicating good affinity and expression. The top 6 rows of logo plots are for monoclonal antibodies; the bottom 5 rows are for polyclonal plasma.

bioRxiv preprint doi: https://doi.org/10.1101/2021.03.17.435863; this version posted March 18, 2021. The copyright holder for this preprint (which was not certified by peer review) is the author/funder, who has granted bioRxiv a license to display the preprint in perpetuity. It is made available under aCC-BY 4.0 International license.

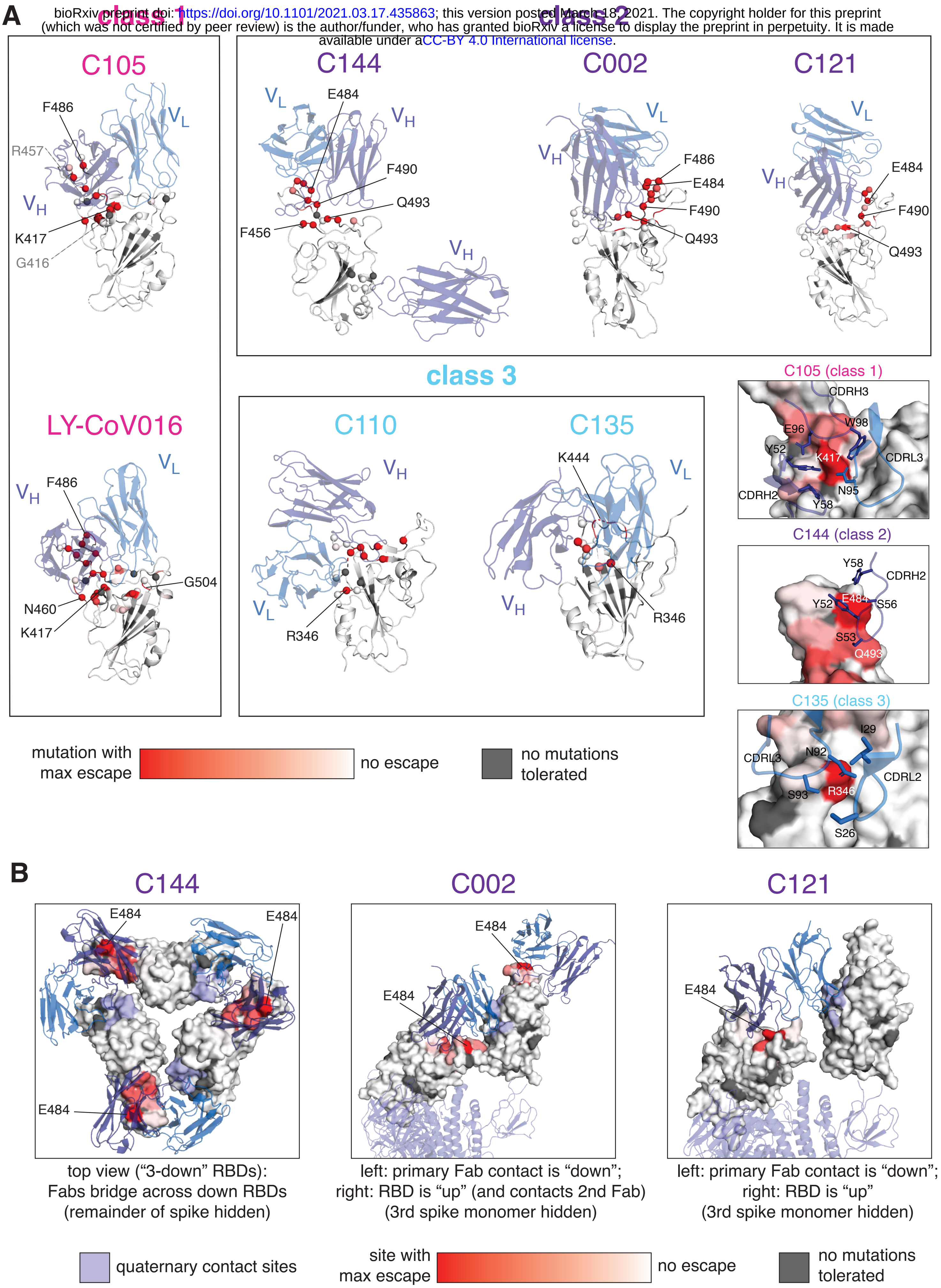

bioRxiv preprint doi: https://doi.org/10.1101/2021.03.17.435863; this version posted March 18, 2021. The copyright holder for this preprint (which was not certified by peer review) is the author/funder, who has granted bioRxiv a license to display the preprint in perpetuity. It is made available under aCC-BY 4.0 International license.

Figure S5. Visualization of the maximum escape at a site mapped onto cartoon representations of antibody-bound RBD, related to Figure 2. (A) Mapping of maximum antibody escape at a site to the antibody-bound RBD. Antibody-contact sites on the RBD (within 4 Å) are shown as spheres. Sites with no escape measurements due to excessive functional constraint on the site are shown in dark gray. Each site is colored according to the maximum escape fraction of any mutation at that site (whereas **Figure 2** shows site total escape), scaled from white (no escape) to red (maximum escape for any mutation for that antibody). Inset panels at right indicate key RBD-antibody interactions where mutations to the indicated RBD site disrupt antibody binding. RBD color scale indicates site total escape, as in **Figure 2A**. **(B)** Visualization of class 2 antibody quaternary epitopes. The total escape at each site is mapped onto the surface of the Fab-bound RBD as in **Figure 2A**, with white indicating no escape, and red indicating the site with the most escape from that antibody. Sites where no mutations are tolerated are indicated in dark gray. Antibody quaternary contact sites are shown in periwinkle. The C144 antibody binds to spike trimer in the "all RBDs down" conformation and forms a quaternary epitope that bridges across two adjacent RBDs by binding to a hydrophobic RBD cavity at the base of the N343 N-linked glycan. The C002 and C121 antibodies, when bound to a "down" RBD, can form a quaternary epitope with an adjacent "up" RBD. The "up" RBD also contacts another C121 Fab[25]. Our yeast-display system utilizes monomeric RBD and therefore does not map escape mutations to quaternary contact sites. These results thus cannot be used to determine the importance of the quaternary sites for antibody binding. Previous work, however, has shown that the V367F RBD mutation to the C144 quaternary epitope does not affect neutralization of pseudotyped lentivirus by C144[7]. See **Methods** for PDB accession codes used to generate structural representations.

bioRxiv preprint doi: https://doi.org/10.1101/2021.03.17.435863; this version posted March 18, 2021. The copyright holder for this preprint (which was not certified by peer review) is the author/funder, who has granted bioRxiv a license to display the preprint in perpetuity. It is made available under aCC-BY 4.0 International license.

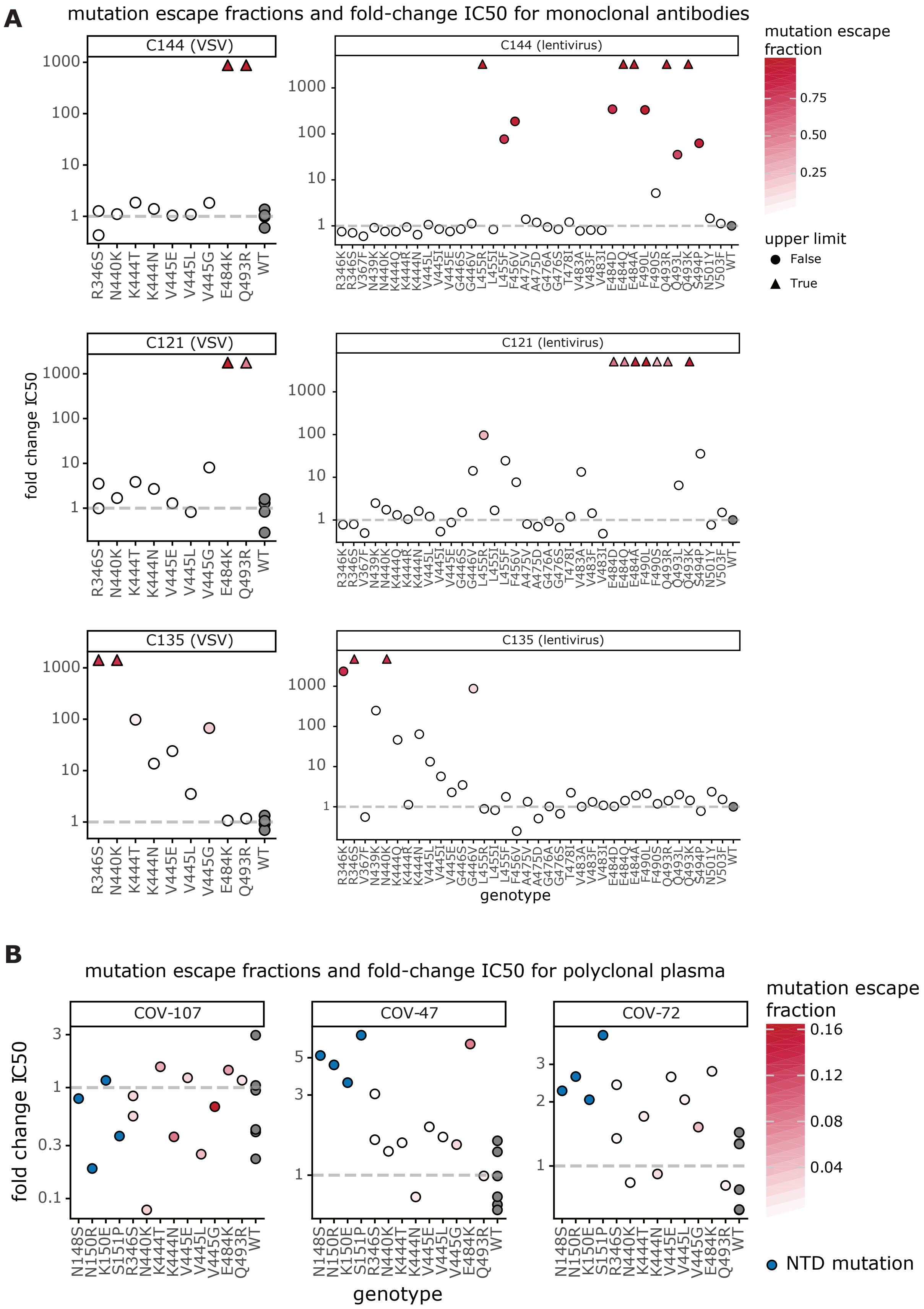

bioRxiv preprint doi: https://doi.org/10.1101/2021.03.17.435863; this version posted March 18, 2021. The copyright holder for this preprint (which was not certified by peer review) is the author/funder, who has granted bioRxiv a license to display the preprint in perpetuity. It is made available under aCC-BY 4.0 International license.

Figure S6. The previously measured effects of spike mutations on neutralization for 3 monoclonal antibodies and 3 polyclonal plasma, related to Figure 4. (A) The effects of mutations on neutralization of chimeric VSV encoding the SARS-CoV-2 (left) or spike-pseudotyped lentivirus particles (right) by monoclonal antibodies from Weisblum et al. (2020)[7]. The y-axis shows the fold-change in IC50 compared to the Wuhan-Hu-1-like spike, such that larger numbers are greater reductions in neutralization sensitivity. Mutations that had IC50s at or above the limit of detection are indicated as triangles. Points are colored according to their mutation escape fraction (**Figure 1, Supplementary Table 1**). Wildtype is in gray. **(B)** The effects of mutations on neutralization of spike-encoding chimeric VSV by polyclonal antibodies from Weisblum et al. (2020)[7]. Wildtype spike IC50 values are shown in gray. NTD mutations are shown in blue. Escape fraction color scales are independent in **(A)** and **(B)**.

bioRxiv preprint doi: https://doi.org/10.1101/2021.03.17.435863; this version posted March 18, 2021. The copyright holder for this preprint (which was not certified by peer review) is the author/funder, who has granted bioRxiv a license to display the preprint in perpetuity. It is made available under aCC-BY 4.0 International license.

Supplemental Files

Supplementary Table 1. Measurements of effects of all amino-acid mutations to the RBD on binding of monoclonal antibodies or polyclonal human plasma, related to Figure 1.

The file gives the "escape fraction" for each mutation, as well as the total escape fraction at each site and the maximum escape fraction for any mutation at the site. The file is also available on GitHub at https://github.com/jbloomlab/SARS-CoV-2-RBD_MAP_Rockefeller/blob/main/results/supp_data/all_samples_raw_data.csv.

Supplementary Table 2. Information on FACS sorting to select cells expressing RBD mutants with reduced binding by antibodies or plasma, related to Figure 1 and Figure S1.

The file gives the number of antibody-escaped cells collected per selection for each replicate library and the percent of RBD+ cells in the antibody-escape gate for each selection, and the exact dilution used for each plasma selection. The file is also available on GitHub at https://github.com/jbloomlab/SARS-CoV-2-RBD_MAP_Rockefeller/blob/main/data/SupplementaryTable2.xlsx.
